# Regional cingulate metabolite-ratio differences exceed longitudinal changes across an approximately 34-h protocol: a prospective quality-controlled proton magnetic resonance spectroscopy study

**DOI:** 10.3389/fmed.2026.1990412

**Published:** 2026-10-28

**Authors:** Sihang Wang, Youquan Ning, Zhiwei Zhang, Bin Yu, Hao Wu, Yilan Li, Lian He, Yida Hu, Fajin Lv

**Affiliations:** 1 Department of Radiology, The First Affiliated Hospital of Chongqing Medical University, Chongqing, China; 2 Department of Neurology, The First Affiliated Hospital of Chongqing Medical University, Chongqing, China

**Keywords:** anterior cingulate cortex, longitudinal change, MEGA-PRESS, polysomnography, posterior cingulate cortex, proton magnetic resonance spectroscopy, quality control, SVS-PRESS

## Abstract

**Background:**

Regional organization, acquisition sequence, and measurement quality can obscure the interpretation of longitudinal proton magnetic resonance spectroscopy (1H-MRS) changes. We compared anterior vs. posterior cingulate metabolite-ratio differences with changes across an approximately 34-h protocol and tested whether findings depended on sequence, quality-control rules, respiratory burden, comorbidity, or polysomnographic sleep measures.

**Methods:**

In this prospective single-center observational study, 138 university-affiliated and advertisement-recruited participants underwent baseline evening spectroscopy of the anterior cingulate cortex (ACC) and posterior cingulate cortex (PCC) on day 1, full-night polysomnography from day 2 into day 3, and post-PSG morning spectroscopy on day 3 using MEGA-PRESS and SVS-PRESS. Among 116 participants with complete paired timestamps, the median interval between scan starts was 34.01 h (IQR, 33.86–34.10 h). Primary metabolite-to-creatine ratios were analyzed using age-, sex-, and body mass index-adjusted generalized estimating equations. Sensitivity analyses evaluated effect magnitude, sequence dependence, spectral-quality selection, follow-up availability, exclusion of participants with apnea-hypopnea index (AHI) ≥15 events/h, exclusion of hypertension or diabetes, and associations between metabolite change and seven polysomnographic measures.

**Results:**

A total of 137 participants contributed to adjusted primary models. PCC showed higher NAA/Cr in both sequences and higher SVS GlxT/Cr, whereas ACC showed higher Cho/Cr in both sequences and higher MEGA Glu/Cr (all *q* < 0.001); PCC also showed higher MEGA GABA/Cr (*q* = 0.020). Cr2 denotes the vendor-fitted second creatine/phosphocreatine resonance, and GlxT denotes the sum of six vendor-fitted glutamate/glutamine components. Follow-up reductions that survived false-discovery-rate correction were confined to SVS Cr2/Cr and GlxT/Cr. Across 11 outcomes, the median absolute paired standardized effect size was 0.571 for region and 0.125 for longitudinal change; the former was 4.6 times the latter, a ratio of median standardized effect magnitudes rather than a biological fold difference. Excluding AHI ≥ 15 events/h preserved the direction of all 12 primary FDR-significant effects, with 11 remaining *q* < 0.05. None of 77 polysomnographic dose-response tests survived global correction.

**Conclusions:**

In this heterogeneous cohort, regional differences in cingulate metabolite ratios were larger and more robust than longitudinal changes. Because the approximately 34-h contrast combined elapsed time, circadian phase, an intervening monitored night, repositioning, and other uncontrolled influences, it should not be interpreted as a sleep-specific effect. The ratio-based findings should not be interpreted as regional concentration differences.

## Introduction

Longitudinal changes in neural activity, synaptic physiology, energy demand, and fluid-related processes may span sleep and wake, but a two-time-point contrast is not a sleep-only manipulation. It can incorporate circadian phase, fasting or feeding interval, time awake before scanning, intervening daytime activity, repositioning, and potential scanner drift (1–4). Repeated, region-resolved measurement with explicit physiological phenotyping and verified timing is therefore needed before a longitudinal difference can be attributed to sleep itself.

The cingulate cortex is well suited to test this principle. The anterior cingulate cortex (ACC) participates in salience detection, arousal regulation, affective control, and performance monitoring, whereas the posterior cingulate cortex (PCC) is a central default-mode-network hub involved in memory integration, self-referential processing, and vigilance-state regulation (5, 6). These functional distinctions have a neurochemical analogue. High-field work demonstrated regionally patterned cingulate GABA, glutamine, and glutamate concentrations, and a recent 3T study showed an average rostral-to-caudal glutamate gradient while also identifying substantial between-person heterogeneity (7, 8). Pooling ACC and PCC may therefore obscure the strongest source of variation.

Proton magnetic resonance spectroscopy (1H-MRS) provides a non-invasive window on this regional chemistry. NAA is commonly interpreted in relation to neuronal and mitochondrial integrity; choline-containing compounds to membrane turnover; creatine and phosphocreatine resonances to energy buffering; myo-inositol to glial-osmotic physiology; glutamate and GABA to excitatory and inhibitory metabolism; and lactate to glycolytic demand (9). These assignments are useful but not one-to-one biological readouts. Localization, echo time, editing, tissue composition, macromolecular co-editing, spectral overlap, signal-to-noise ratio (SNR), linewidth, preprocessing, fitting, and reference choice all influence the estimate (10–14).

Prior sleep-MRS findings illustrate both the promise and the inconsistency of the field. Shorter sleep duration and primary insomnia have been linked to altered GABAergic signals (15–17). Repeated morning-evening studies have not consistently confirmed a uniform diurnal GABA effect, while developmental and adult studies of Glx have reported region- and age-dependent changes (18–20). Obstructive sleep apnea has likewise been associated with altered GABA and glutamate (21). Systematic reviews emphasize that MRS measures of GABA and glutamate should not be treated as direct measures of synaptic transmission or functional excitation-inhibition balance (22–24).

Three questions motivated the present study. First, how much larger are ACC-PCC metabolite-ratio differences than baseline-to-follow-up changes across an approximately 34-h protocol on a common within-participant scale? Second, do shared metabolites support cross-sequence pooling, or does acquisition sequence alter regional and temporal estimates? Third, are the conclusions robust to spectral-quality rules, selective follow-up availability, respiratory burden, common recorded medical comorbidities, and variation in polysomnographic sleep architecture or quality? We addressed these questions in a prospective paired cingulate-MRS cohort. The primary hypothesis was that regional metabolite-ratio organization would exceed a uniform longitudinal effect. Clinical phenotype associations remained exploratory and are reported in the supplement rather than framed as biomarker validation.

## Materials and methods

### Study design and participants

This prospective single-center observational study used a standardized three-calendar-day workflow. Participants completed baseline assessment, questionnaires, baseline MRI/MRS, full-night polysomnography (PSG), post-PSG MRI/MRS, and next-morning memory assessment. Participants were recruited through Chongqing Medical University and The First Affiliated Hospital of Chongqing Medical University and through study advertisements between May 2024 and September 2025. The 138-person analysis cohort included university students and trainees as well as community or working volunteers. Aggregate educational records comprised 11 participants in a junior-college category, 70 in an undergraduate or bachelor's-level category, 41 in a master's-level category, and 16 in a doctoral-level category. These educational categories did not map one-to-one to recruitment source, occupation, internship status, or night-shift exposure. Among 150 people screened, 12 were excluded because of organic brain lesions, a relevant medication-use history, MRI contraindications, or other protocol criteria, leaving 138 enrolled participants. Reason-specific exclusion counts were not retained. Eligible participants were aged 18 years or older and right-handed. Exclusion criteria comprised a previously diagnosed sleep disorder; neurologic, psychiatric, or serious medical illness; organic brain disease; drug dependence; a family history of psychosis; a history of psychotropic, hypnotic, or antidepressant use; and MRI contraindications. All enrolled participants provided written informed consent. Fourth-year interns were not assigned night-shift rotations; however, the 70 participants in the aggregate undergraduate or bachelor's-level category were not all 4-year interns and included some community or working volunteers recruited through study advertisements. Individual internship status and night-shift or on-call exposure were not systematically recorded for the full cohort. Duty exposure was therefore not used as a covariate or subgroup factor, and no duty-status comparison was performed. Participants with a previously diagnosed sleep disorder were excluded, but PSG performed after enrollment identified a heterogeneous range of previously unrecognized sleep-disordered-breathing burden; the cohort should therefore not be interpreted as uniformly healthy or as a single-diagnosis sample. The analysis cohort comprised 138 participants with a de-identified study identifier and at least one finite MRS ratio. No formal *a priori* sample-size calculation was performed; the sample was determined by completed protocol records with analyzable spectroscopy. Adjusted models additionally required age, sex, and body mass index (BMI). BMI was absent in the source field for 12 participants and was reconstructed exactly from recorded weight divided by height squared for 11, leaving one participant unavailable for adjusted models; this was an anthropometric derivation, not statistical imputation. The Medical Research Ethics Review Committee of The First Affiliated Hospital of Chongqing Medical University approved the protocol (No. 2024-145-01). The study followed the Declaration of Helsinki.

### Study procedure and phenotyping

Participants followed a three-calendar-day protocol (Figure 1). On the evening of day 1, participants completed baseline assessment and baseline MRI/MRS while awake. Full-night PSG was initiated on the evening of day 2 and continued into the morning of day 3, after which post-PSG MRI/MRS and memory assessment were performed. Scanner-derived dates, start times, and end times were available for 133 baseline examinations and 120 post-PSG examinations; complete paired timing records were available for 116 of 138 participants. Among these 116 participants, all post-PSG examinations occurred two calendar days after the corresponding baseline examination. The median start time was 21:05 (IQR, 21:01–21:12) for the baseline examination and 07:05 (IQR, 07:01–07:09) for the post-PSG examination. The median interval between scan starts was 34.01 h (IQR, 33.86–34.10; range, 33.34–34.43 h). Missing timestamps were not imputed. The labels “pre” and “post” are retained in variable names to match the prespecified data fields and denote baseline and post-PSG follow-up, respectively; they do not denote an immediate same-night pre-/post-PSG comparison. This schedule was selected for pragmatic and participant-preparation reasons. Scheduling baseline research MRI/MRS one evening earlier allowed the subsequent PSG night to provide an adequate recording and sleep opportunity without compressing pre-bed preparation, while completing the post-PSG scan before 08:00 avoided displacing routine clinical examinations on the shared scanner. Separating baseline MRI/MRS from the PSG night may also have reduced immediate burden before the PSG night from travel, preparation, and scanner exposure; this was an operational design consideration, not evidence that the intervening day was biologically inert. Participants were instructed to avoid coffee, tea, alcohol, and daytime naps on the monitoring day and to limit evening fluid intake. PSG variables included total sleep time (TST), sleep efficiency, wake after sleep onset (WASO), sleep-onset latency, sleep-stage durations and proportions, arousal index, apnea-hypopnea index (AHI), oxygen desaturation index, oxygen desaturation burden, and periodic limb movement index, scored using contemporaneous American Academy of Sleep Medicine criteria (25). Questionnaires included the Pittsburgh Sleep Quality Index, Epworth Sleepiness Scale, STOP-BANG, Morningness-Eveningness Questionnaire, Self-Rating Anxiety Scale, Self-Rating Depression Scale (SDS), Montreal Cognitive Assessment, and Wechsler Memory Scale (26–33). Habit variables were coded from the source workbook as smoking (never/former/current), alcohol use (none/<3 times per week/≥3 times per week), exercise (none/<150 min per week/≥150 min per week), and coffee or tea use (none/occasional/frequent). These variables characterized the cohort and supported exploratory analyses; they did not define a single disease group.

**Figure 1:**
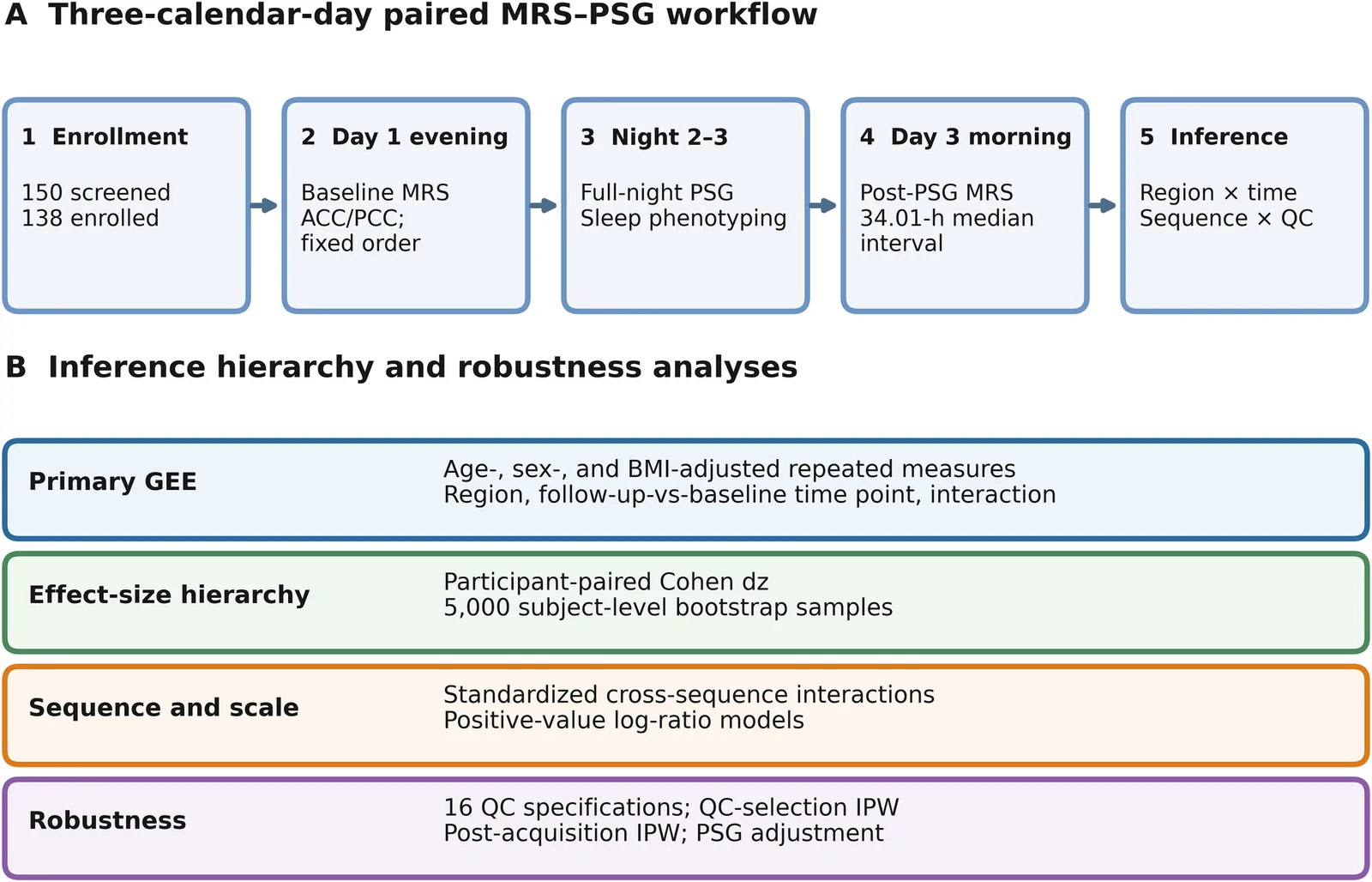
Three-calendar-day paired cingulate-MRS workflow and inference hierarchy. **(A)** Summarizes 150 people screened, 12 excluded, and 138 enrolled, followed by day 1 evening baseline MRS, day 2-to-3 full-night PSG, and day 3 morning post-PSG MRS. Among 116 participants with paired timestamps, the median interval between scan starts was 34.01 h. **(B)** Summarizes the primary GEE and complementary magnitude, sequence, scale, quality, availability, AHI/comorbidity, and PSG dose-response analyses.

### Magnetic resonance acquisition

Imaging was performed on a United Imaging uMR 790 3.0 T system (uExceed R003; full version R003.1.5.1628933-CD11P5; application platform V10_3T_R3) with an integrated volume transmit coil and a 32-channel head receive coil. Foam padding was used to reduce head motion. The system specified a maximum gradient strength of 100 mT/m and maximum slew rate of 200 mT/m/ms. Three-dimensional T1-weighted imaging was used for localization (TR 3 ms, TE 2.3 ms, field of view 256 mm × 256 mm, matrix 256 × 256, slice thickness 1.0 mm, bandwidth 260 Hz, echo-train length 252, NEX 1). Single 20 mm × 20 mm × 20 mm voxels were centered on the midline and checked in sagittal, axial, and coronal views. The ACC voxel was positioned above the genu of the corpus callosum with approximately 2-mm clearance from the callosal margin. The PCC voxel followed the posterior splenial border with approximately 2-mm clearance from the splenium and adjacent occipitoparietal fissure. The same operator used the baseline orthogonal views as visual landmarks for post-PSG repositioning; quantitative overlap maps were not available. At both the baseline and post-PSG sessions, the fixed acquisition order was ACC SVS-PRESS, ACC MEGA-PRESS, PCC SVS-PRESS, and PCC MEGA-PRESS. SVS-PRESS (vendor sequence svs_press; protocol svs_press_te144_1) used TR 2,000 ms, TE 144 ms, bandwidth 1,000 Hz, 1,024 sampling points, NEX 128, four-step phase cycling, a 2.0-ppm chemical-shift setting, spectroscopy shimming, strong water suppression with 96-Hz bandwidth, dynamic frequency calibration, and a scan time of 4 min 19 s. MEGA-PRESS (vendor sequence svs_mega; protocol mega-mms_1) used TR 2,000 ms, TE 69 ms, bandwidth 1,000 Hz, 1,024 points, NEX 160, two-step phase cycling, a 3.0-ppm chemical-shift setting, edit and reference frequencies of 1.90 and 1.50 ppm, spectroscopy shimming, dynamic frequency calibration, and a scan time of 10 min 48 s. The symmetric 1.90/1.50-ppm scheme was designed to suppress co-edited macromolecular signal around 3 ppm; because editing-pulse bandwidth/duration and transient-level frequency traces were unavailable, the output is described as nominally macromolecule-suppressed GABA rather than a macromolecule-free measurement (34, 35). Representative placements and spectra are shown in Figure 2.

**Figure 2:**
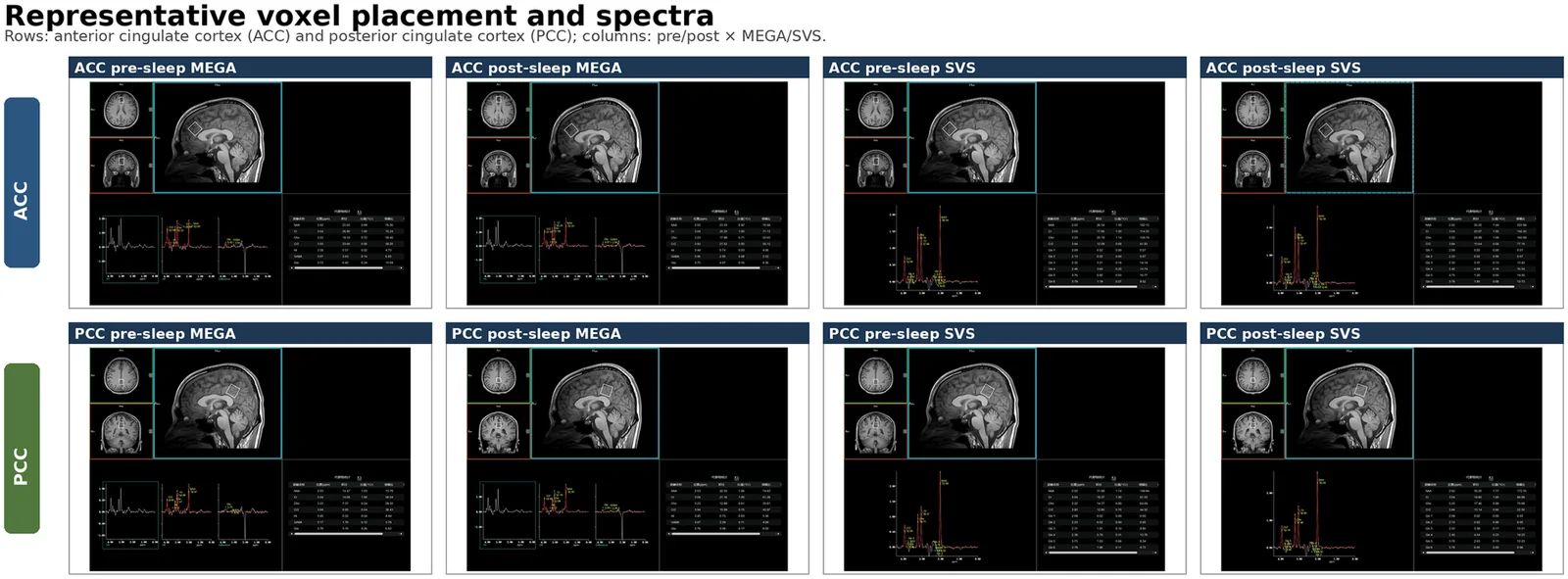
Representative voxel placement and spectra across regions, time points, and sequences. De-identified structural images show ACC and PCC localization with representative baseline and post-PSG MEGA-PRESS and SVS-PRESS spectra. Displayed spectra are illustrative and are not substitutes for cohort-level quality metrics.

### Spectral processing and relative quantification

Spectra were processed on the manufacturer-provided uWS-MR workstation (version 2.1; United Imaging Healthcare) using the integrated spectroscopy analysis module. Presets Brain_SVS_3T_SE144_1 and Brain_SVS_3T_Mega_SE68 were used for SVS-PRESS and MEGA-PRESS, respectively; the latter preset name did not override the acquired TE of 69 ms recorded in the protocol. Retained workstation documentation supported automatic Gaussian peak fitting. SVS outputs were derived from the conventional PRESS spectrum, whereas the MEGA preset reconstructed edited and reference acquisitions and generated a difference spectrum for the GABA and vendor-labeled Glu outputs. The software export contained peak position, integral, signal-to-noise ratio (SNR), full width at half maximum (FWHM), and metabolite-to-Cr ratios. A separate unsuppressed-water acquisition was not present in the retained study files, so water-referenced absolute concentrations could not be verified or calculated from the supplied archive. Other preprocessing settings, basis functions, constraints, baseline model, and fit residuals were not available for independent verification.

### Spectroscopy outcomes and spectral quality

The primary outcomes were within-sequence metabolite-to-creatine ratios: MEGA-PRESS NAA/Cr, Cho/Cr, Cr2/Cr, mI/Cr, GABA/Cr, and Glu/Cr; and SVS-PRESS NAA/Cr, Cho/Cr, Cr2/Cr, GlxT/Cr, and Lac/Cr. Cr2 is the vendor-fitted second creatine/phosphocreatine resonance near 3.94 ppm and is distinct from the principal Cr reference near 3.0 ppm. SVS GlxT is the total Glx output, numerically equal to the sum of six vendor-fitted glutamate/glutamine components (Glx1-Glx6). MEGA Glu is retained as the vendor label for an edited-spectrum output; because glutamine overlap could not be separated from the retained export, it should not be read as a chemically pure glutamate concentration. GABA denotes the nominally macromolecule-suppressed edited output defined above. Finite zero-valued outputs were retained on the raw scale, including exact Lac/Cr zeros. Secondary ratios using NAA or Cr2 as the denominator and prespecified within-sequence axes were calculated only when both components were available and were interpreted as relative indices, not absolute concentrations or direct synaptic measures. Spectral quality was summarized using exported SNR, FWHM, chemical-shift position, and analyzable retention. The reference QC rule required a finite ratio, numerator SNR ≥ 3 and FWHM ≤13 Hz, and Cr SNR ≥ 3 and FWHM ≤ 13 Hz. GlxT lacked a metabolite-specific SNR/FWHM field and was not included in threshold-multiverse analyses. These thresholds were operational sensitivity rules rather than universal validity cutoffs (34–43).

### Primary statistical model and multiplicity control

Repeated observations were analyzed using Gaussian generalized estimating equations (GEE) with an exchangeable working correlation and participant-clustered robust standard errors (44). Each sequence-metabolite model included region (PCC = 1, ACC = 0), acquisition time point (post-PSG follow-up = 1, baseline = 0), their interaction, age, sex, and BMI. The region coefficient represented PCC minus ACC at the baseline time point; the time-point coefficient represented post-PSG follow-up minus baseline in ACC; and the interaction represented the differential time-point change in PCC. False-discovery rate (FDR) was controlled with the Benjamini–Hochberg procedure across all region, time-point, and interaction tests within each sequence (18 MEGA-PRESS and 15 SVS-PRESS hypotheses) (45). Two-sided *q* < 0.05 defined statistical significance for the primary family.

### Magnitude, sequence, scale, and quality robustness analyses

To place region and longitudinal effects on a common scale, participant-level PCC-minus-ACC differences were averaged across available time points and post-pre differences were averaged across available regions for each of 11 outcomes. Paired Cohen dz and percentile bootstrap confidence intervals were calculated. The hierarchy statistic was the median absolute regional dz minus the median absolute longitudinal dz across outcomes; its 95% confidence interval used 5,000 participant-level bootstrap samples. A 90% interval entirely within dz = −0.20 to 0.20 was used only as a supplementary equivalence sensitivity analysis. Positive-value log-ratio models asked whether conclusions depended on additive rather than proportional scale. Combined standardized models for NAA, Cho, and Cr2 tested whether sequence changed the regional or temporal effect. These analyses addressed effect magnitude, scale, and cross-sequence comparability, respectively, rather than serving as repeated tests of the same hypothesis.

### Robustness to QC selection and follow-up attrition

A QC multiverse crossed numerator and Cr SNR cutoffs of 2, 3, 4, and 5 with FWHM cutoffs of 10, 12, 13, and 15 Hz (16 specifications) to assess dependence on operational spectral-quality rules (46). Inverse-probability weighting separately evaluated selection from the reference QC rule and from unavailable post-PSG acquisitions (47). The primary GEE models were also repeated after excluding participants with AHI ≥ 15 events/h, after excluding recorded hypertension or diabetes, and after applying both restrictions. Because psychotropic, hypnotic, and antidepressant use was an exclusion criterion, no within-cohort medication-restricted sensitivity analysis was performed. To test polysomnographic dose-response rather than merely adjust for PSG, participant-level post-pre change was averaged across regions and regressed separately on TST, sleep efficiency, WASO, slow-wave-sleep duration, REM duration, log1p(arousal index), and log1p(AHI), with age, sex, and BMI adjustment and HC3 standard errors. FDR was controlled across all 77 outcome-exposure tests and, secondarily, across 14 focused tests for SVS Cr2/Cr and GlxT/Cr. Detailed log-ratio, interaction, QC-multiverse, weighting, restriction, and PSG dose-response results are reported in Supplementary Tables S28–S37.

### Computational implementation and reproducibility

Analyses were performed in Python 3.12.13 using NumPy 2.3.5, pandas 2.2.3, SciPy 1.17.0, and scikit-learn 1.8.0. Gaussian GEE and participant-clustered sandwich estimators were implemented by direct matrix algebra following Liang and Zeger (44). Bootstrap analyses used a fixed random seed (20260831). As a reproducibility check, the complete analysis pipeline was rerun from the de-identified export and reproduced the primary point estimates and the updated quality-control outputs from the final de-identified export. Source-workbook names were excluded before analysis and are absent from the submission files.

### Exploratory analyses and reporting

Alternative-denominator models, derived axes, subgroup contrasts, partial Spearman correlations, cross-validated classification screens, cognitive models, and habit associations are reported in the supplementary workbook. Because these analyses span many correlated endpoints and were not externally validated, they are interpreted as hypothesis-generating. Duty status was not analyzed. Fourth-year interns were not assigned night-shift rotations, but the aggregate undergraduate or bachelor's-level category also included community or working volunteers; individual internship status and night-shift or on-call history were not systematically recorded for the full cohort, and no individual duty-frequency or recent-duty-timing variable existed. We did not infer duty exposure from education or recruitment category. During cleaning, SDS entries recorded on a 0–1 scale were multiplied by 100 to restore the raw-score scale, and one impossible MoCA value above the valid 0–30 range was set to missing; these rules and affected counts are documented in Supplementary Table S26. Reporting follows STROBE and MRSinMRS principles (13, 48). Analyses used de-identified copies; the name field present in the source workbook was excluded before analysis and is absent from all submission files.

## Results

### Cohort and data availability

Of 150 people screened, 12 were excluded for organic brain lesions, a relevant medication-use history, MRI contraindications, or other protocol criteria, leaving 138 enrolled participants who provided written informed consent. The source dataset contained 138 unique participants (73 men and 65 women; mean age 33.66 years). Aggregate educational records comprised 11 participants in a junior-college category (8.0%), 70 in an undergraduate or bachelor's-level category (50.7%), 41 in a master's-level category (29.7%), and 16 in a doctoral-level category (11.6%). The undergraduate or bachelor's-level category included both 4-year interns and community or working volunteers recruited through advertisements; it was not an occupational or duty-status category. Individual internship status and night-shift or on-call exposure were not systematically recorded for the full cohort, so duty exposure could not be assigned at participant level and no duty-stratified results are presented. AHI was available for 133: 80 (60.2%) had AHI < 5 events/h, 23 (17.3%) had 5–14.9, 6 (4.5%) had 15–29.9, and 24 (18.0%) had ≥30. Thus, PSG detected a substantial previously unrecognized sleep-disordered-breathing subgroup despite exclusion of known sleep disorders. Baseline MRS was available for 136 participants, any post-PSG MRS for 126, and 137 had age, sex, BMI, and at least one valid primary outcome for adjusted GEE models. Missingness ranged from 3 to 10 participants across questionnaires and from 5 to 12 across the displayed PSG variables. Participants without any post-PSG acquisition were older and had higher BMI and AHI than those with post-PSG data (absolute standardized mean differences 1.15, 0.61, and 1.45), motivating post-availability weighting. Overall and sex-specific summaries, AHI categories, and missing counts are reported in Table 1.

**Table 1:** Overall and sex-specific cohort characteristics, AHI categories, and missingness.

Variable	Overall	Women (*n* = 65)	Men (*n* = 73)	Missing
Age, year	33.66 ± 12.52	31.92 ± 13.68	35.21 ± 11.26	0
BMI, kg/m^2^	23.53 ± 3.62	21.59 ± 2.84	25.29 ± 3.35	1
PSQI	6.84 ± 3.43	6.87 ± 3.96	6.82 ± 2.90	9
ESS	10.02 ± 4.15	10.45 ± 4.34	9.60 ± 3.94	5
STOP-BANG	1.99 ± 1.67	0.97 ± 1.06	2.94 ± 1.57	3
MEQ	51.76 ± 10.54	51.48 ± 10.69	52.03 ± 10.47	6
SAS raw score	30.58 ± 5.37	30.69 ± 5.60	30.47 ± 5.19	3
SDS corrected raw score	32.13 ± 6.11	32.29 ± 5.95	31.99 ± 6.28	3
MoCA	26.72 ± 2.69	27.06 ± 2.88	26.41 ± 2.49	6
WMS	113.73 ± 13.29	113.83 ± 13.26	113.63 ± 13.43	10
TST, min	364.00 ± 70.78	365.47 ± 61.63	362.71 ± 78.40	12
Sleep efficiency, %	84.08 ± 12.88	84.82 ± 10.56	83.41 ± 14.70	11
AHI, events/h	12.12 ± 19.33	4.60 ± 11.88	18.69 ± 22.09	5
ODI, events/h	5.70 ± 10.33	2.44 ± 6.48	8.66 ± 12.18	12
Current smoking	21/137 (15.3%)	1/65 (1.5%)	20/72 (27.8%)	1
Frequent alcohol use (code 2)	6/136 (4.4%)	1/65 (1.5%)	5/71 (7.0%)	2
Frequent coffee use	27/136 (19.9%)	13/65 (20.0%)	14/71 (19.7%)	2
Frequent tea use	35/137 (25.5%)	9/65 (13.8%)	26/72 (36.1%)	1
Hypertension	12/138 (8.7%)	3/65 (4.6%)	9/73 (12.3%)	0
Diabetes mellitus	4/138 (2.9%)	2/65 (3.1%)	2/73 (2.7%)	0
AHI <5 events/h	80/133 (60.2%)	52/62 (83.9%)	28/71 (39.4%)	5
AHI 5–14.9 events/h	23/133 (17.3%)	6/62 (9.7%)	17/71 (23.9%)	5
AHI 15–29.9 events/h	6/133 (4.5%)	0/62 (0.0%)	6/71 (8.5%)	5
AHI ≥30 events/h	24/133 (18.0%)	4/62 (6.5%)	20/71 (28.2%)	5

Continuous variables are mean ± SD; categorical variables are *n*/*N* (%). AHI, apnea-hypopnea index; BMI, body mass index; ESS, Epworth Sleepiness Scale; MEQ, Morningness-Eveningness Questionnaire; MoCA, Montreal Cognitive Assessment; ODI, oxygen desaturation index; PSQI, Pittsburgh Sleep Quality Index; SAS, Self-Rating Anxiety Scale; SDS, Self-Rating Depression Scale; TST, total sleep time; WMS, Wechsler Memory Scale. SDS values stored on a 0–1 scale were multiplied by 100 to restore the raw-score scale. Denominators vary because of item-level missingness.

### Primary regional and time-point effects

Regional effects dominated the primary models (Table 2; Figure 3). PCC had higher MEGA NAA/Cr (beta = 0.093, 95% CI: 0.058–0.128, *q* < 0.001) and GABA/Cr (beta = 0.022, 95% CI: 0.007–0.037, *q* = 0.020), whereas ACC had higher MEGA Cho/Cr (beta = −0.122, 95% CI: −0.136 to −0.109, *q* < 0.001) and Glu/Cr (beta = −0.051, 95% CI: −0.067 to −0.034, *q* < 0.001). SVS-PRESS showed higher PCC NAA/Cr (beta = 0.175, 95% CI: 0.158–0.192, *q* < 0.001), GlxT/Cr (beta = 0.064, 95% CI: 0.051–0.077, *q* < 0.001), and Lac/Cr (beta = 0.027, 95% CI: 0.019–0.036, *q* < 0.001), with higher ACC Cho/Cr (beta = −0.210, 95% CI: −0.223 to −0.196, *q* < 0.001). FDR-retained time-point effects were limited to post-PSG follow-up reductions in SVS Cr2/Cr (beta = −0.015, 95% CI: −0.024 to −0.006, *q* = 0.003) and SVS GlxT/Cr (beta = −0.033, 95% CI: −0.044 to −0.021, *q* < 0.001). MEGA Cho/Cr and GABA/Cr had modest region-by-time-point interactions (*q* = 0.041 for each).

**Table 2:** Primary age-, sex-, and BMI-adjusted generalized estimating equation models.

Outcome	*n*	Region beta (95% CI)	*q*	Time-point beta (95% CI)	*q*	Interaction beta (95% CI)	*q*
MEGA NAA/Cr	137	0.093 (0.058 to 0.128)	<0.001	0.001 (−0.032 to 0.035)	0.931	0.003 (−0.045 to 0.051)	0.931
MEGA Cho/Cr	137	−0.122 (−0.136 to −0.109)	<0.001	−0.003 (−0.017 to 0.010)	0.818	0.023 (0.005 to 0.042)	0.041
MEGA Cr2/Cr	137	−0.024 (−0.046 to −0.003)	0.062	−0.011 (−0.035 to 0.014)	0.595	0.003 (−0.030 to 0.035)	0.931
MEGA mI/Cr	137	0.006 (−0.005 to 0.017)	0.475	−0.001 (−0.012 to 0.010)	0.931	−0.005 (−0.019 to 0.008)	0.602
MEGA GABA/Cr	137	0.022 (0.007 to 0.037)	0.020	−0.009 (−0.021 to 0.003)	0.296	0.026 (0.005 to 0.047)	0.041
MEGA Glu/Cr	137	−0.051 (−0.067 to −0.034)	<0.001	−0.017 (−0.032 to −0.002)	0.062	−0.008 (−0.028 to 0.011)	0.595
SVS NAA/Cr	137	0.175 (0.158 to 0.192)	<0.001	0.006 (−0.008 to 0.020)	0.533	−0.001 (−0.019 to 0.017)	0.914
SVS Cho/Cr	137	−0.210 (−0.223 to −0.196)	<0.001	0.003 (−0.008 to 0.014)	0.699	0.007 (−0.006 to 0.021)	0.446
SVS Cr2/Cr	137	0.011 (0.001 to 0.021)	0.060	−0.015 (−0.024 to −0.006)	0.003	−0.004 (−0.015 to 0.007)	0.597
SVS GlxT/Cr	137	0.064 (0.051 to 0.077)	<0.001	−0.033 (−0.044 to −0.021)	<0.001	−0.019 (−0.039 to 0.000)	0.097
SVS Lac/Cr	136	0.027 (0.019 to 0.036)	<0.001	−0.004 (−0.011 to 0.003)	0.440	−0.001 (−0.011 to 0.008)	0.826

Region is PCC minus ACC; time point is post-PSG follow-up minus baseline in ACC; interaction is the differential post-pre change in PCC. FDR was controlled across all 18 MEGA-PRESS or 15 SVS-PRESS primary contrasts within sequence. Participant *n* was 137 except SVS Lac/Cr (*n* = 136).

**Figure 3:**
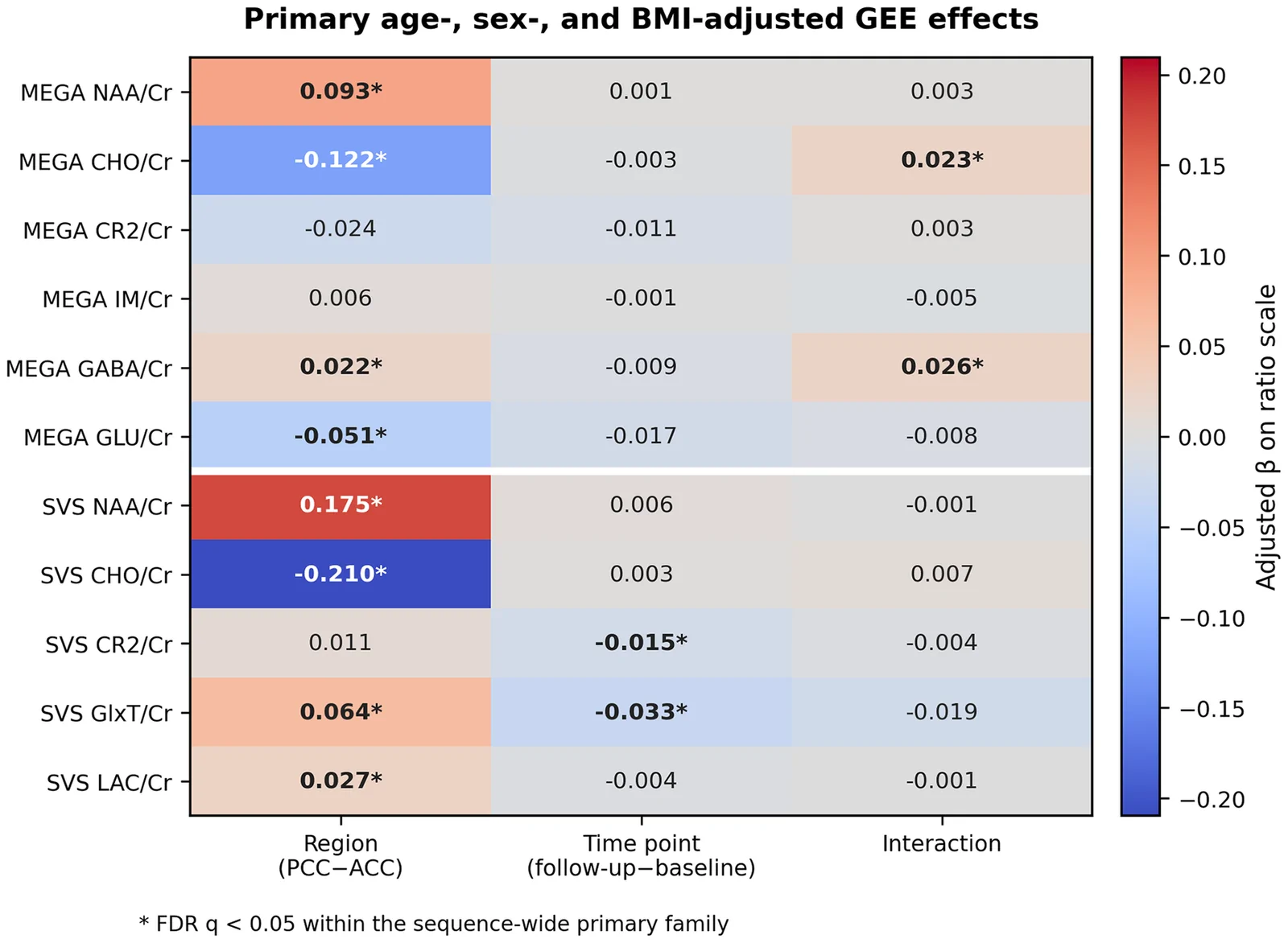
Primary adjusted effects. Heatmap values are GEE coefficients on the original metabolite-to-creatine ratio scale. Region is PCC minus ACC, time point is post-PSG follow-up minus baseline in ACC, and interaction is the differential post-pre change in PCC. Asterisks indicate sequence-wide FDR *q* < 0.05. Participant *n* = 137 except SVS Lac/Cr (*n* = 136).

### Direct effect-magnitude hierarchy

Participant-paired standardized contrasts quantified the central hierarchy (Table 3; Figure 4). Across the 11 outcomes, median absolute dz was 0.571 for regional contrasts and 0.125 for longitudinal contrasts. The regional median was therefore 4.6 times the longitudinal median; this compares standardized effect-size summaries and is not a 4.6-fold difference in metabolite concentration or biological magnitude. Their absolute difference was 0.446 (95% bootstrap CI: 0.324–0.609), and the regional median exceeded the temporal median in all 5,000 bootstrap samples. The largest regional effects were SVS Cho/Cr (dz = −2.934), SVS NAA/Cr (dz = 1.773), and MEGA Cho/Cr (dz = −1.724). The largest temporal effects were SVS GlxT/Cr (dz = −0.731) and SVS Cr2/Cr (dz = −0.509). Only two of 11 temporal outcomes met the supplementary equivalence criterion; nonsignificant time-point terms were not interpreted as proof of no meaningful change.

**Table 3:** Effect-magnitude hierarchy and formal cross-sequence regional interactions.

Analysis	Contrast	Estimate	95% CI	q	Interpretation
Magnitude hierarchy	Median absolute regional dz	0.571	–	–	Across 11 outcomes
Magnitude hierarchy	Median absolute longitudinal dz	0.125	–	–	Across 11 outcomes
Magnitude hierarchy	Regional minus longitudinal median	0.446	0.324 to 0.609	–	5,000 participant bootstraps
Sequence interaction	NAA: sequence × region	0.575	0.337 to 0.813	<0.001	Standardized within sequence
Sequence interaction	CHO: sequence × region	−0.163	−0.307 to −0.019	0.079	Standardized within sequence
Sequence interaction	CR2: sequence × region	0.465	0.178 to 0.752	0.007	Standardized within sequence

**Figure 4:**
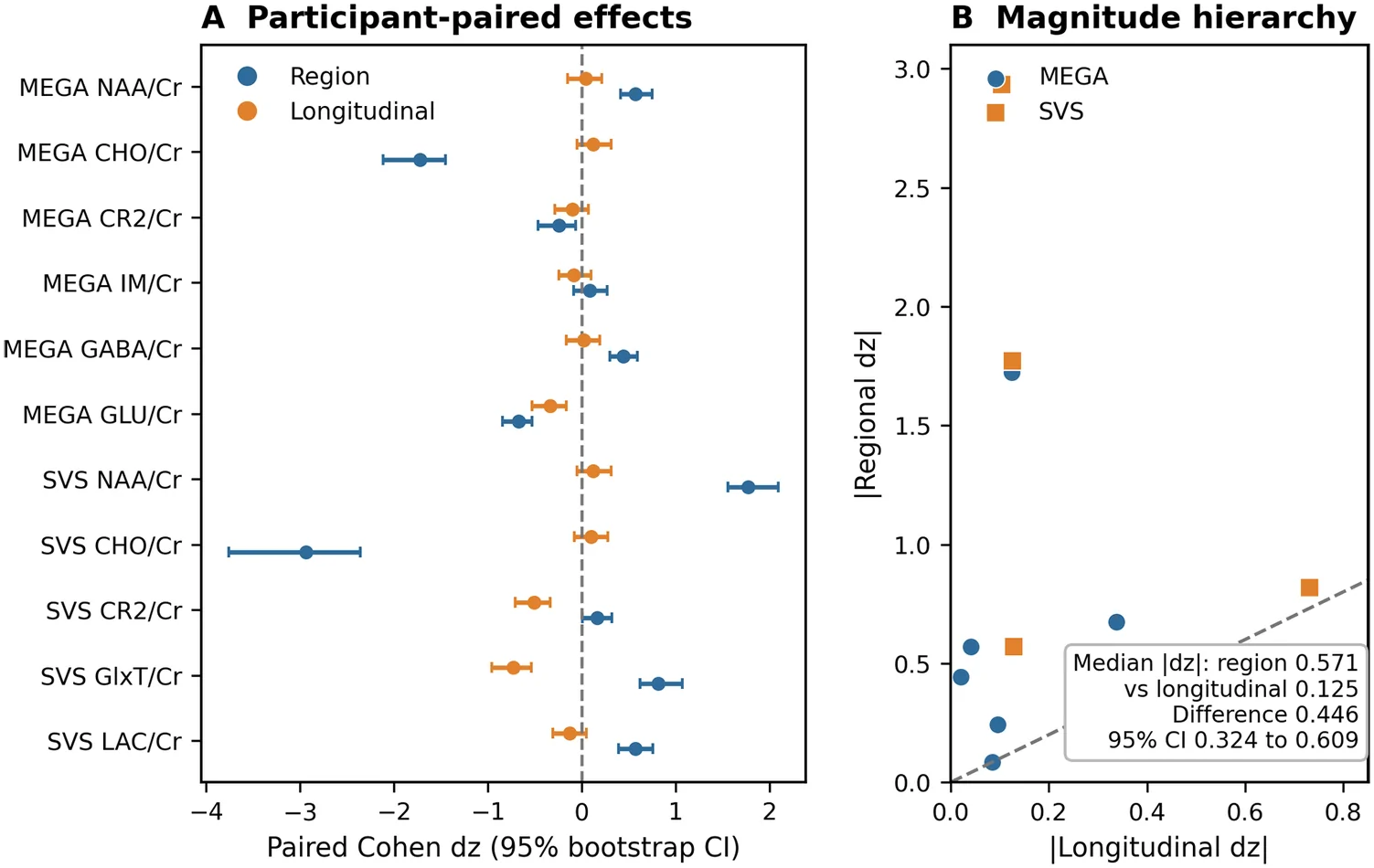
Direct regional-vs.-longitudinal effect hierarchy. **(A)** Shows participant-paired Cohen dz values with 95% bootstrap confidence intervals. **(B)** Plots absolute regional and temporal effect sizes; points above the diagonal have larger regional effects. Regional contrasts used 136–138 pairs and temporal contrasts 120–124 pairs. The inset reports the median hierarchy and its 5,000-sample bootstrap interval.

### Sequence and scale sensitivity

NAA and Cho retained the same regional direction in both sequences, but formal standardized models showed that effect magnitude depended on sequence (Figure 5). Sequence-by-region interactions survived correction for NAA (standardized beta = 0.575, 95% CI: 0.337–0.813, *q* < 0.001) and Cr2 (beta = 0.465, 95% CI: 0.178–0.752, *q* = 0.007); the Cho interaction did not survive the nine-test correction (*q* = 0.079). No sequence-by-time-point or three-way interaction survived FDR correction. Positive-value log-ratio models preserved the main regional directions and estimated post-PSG SVS Cr2/Cr and GlxT/Cr values as 2.7% and 7.3% lower, respectively. Detailed proportional estimates are provided in Supplementary Table S28; log models excluded non-positive values and were sensitivity analyses rather than replacements for the raw-scale primary models. Sequence-specific spectral-quality profiles are summarized in Figure 6.

**Figure 5:**
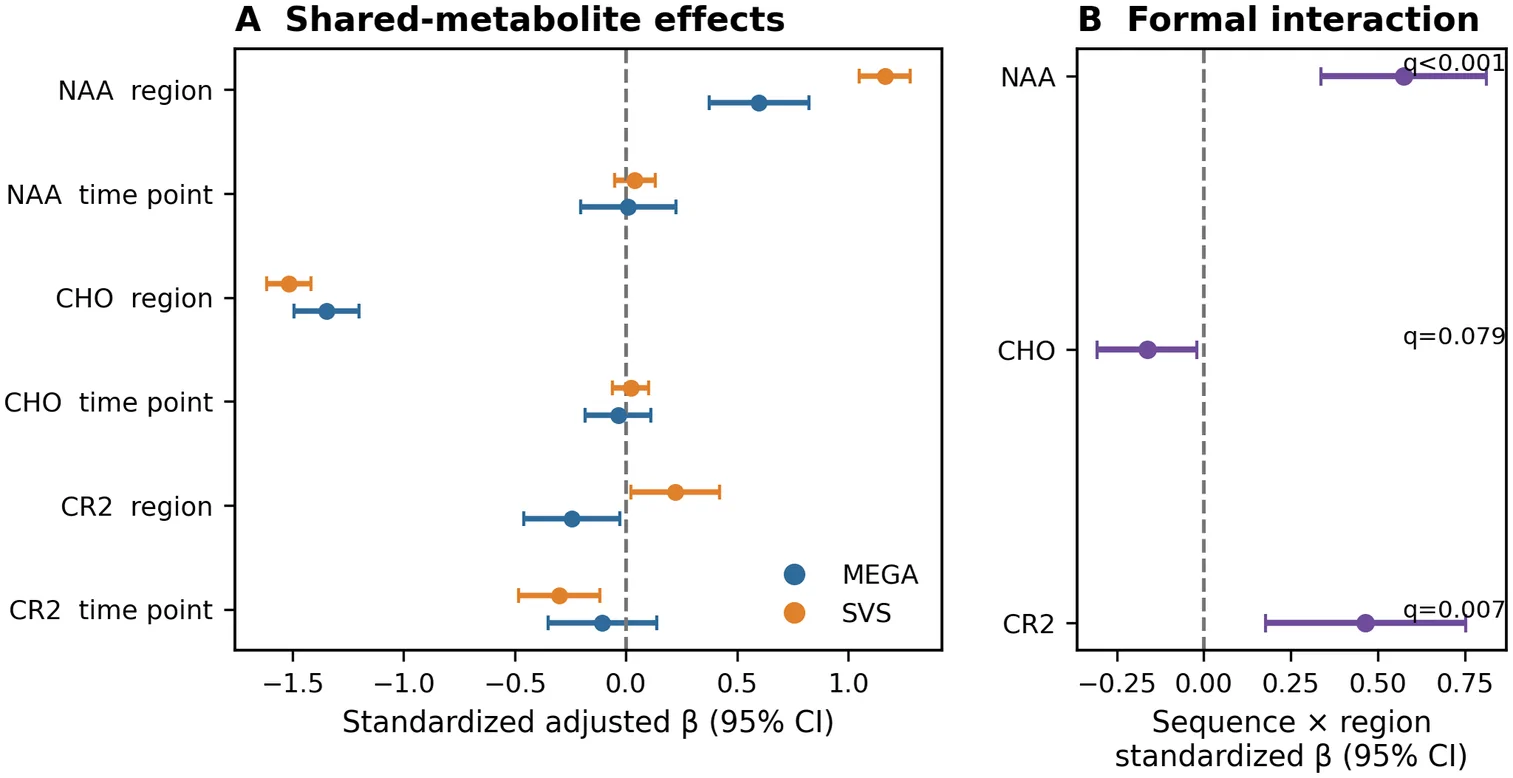
Sequence dependence of shared metabolites. **(A)** Compares standardized region and time-point coefficients for NAA, Cho, and Cr2 across MEGA-PRESS and SVS-PRESS. **(B)** Shows formal standardized sequence-by-region interaction estimates with 95% confidence intervals and FDR *q* values (*n* = 137; 1,026 observations per shared-metabolite model).

**Figure 6:**
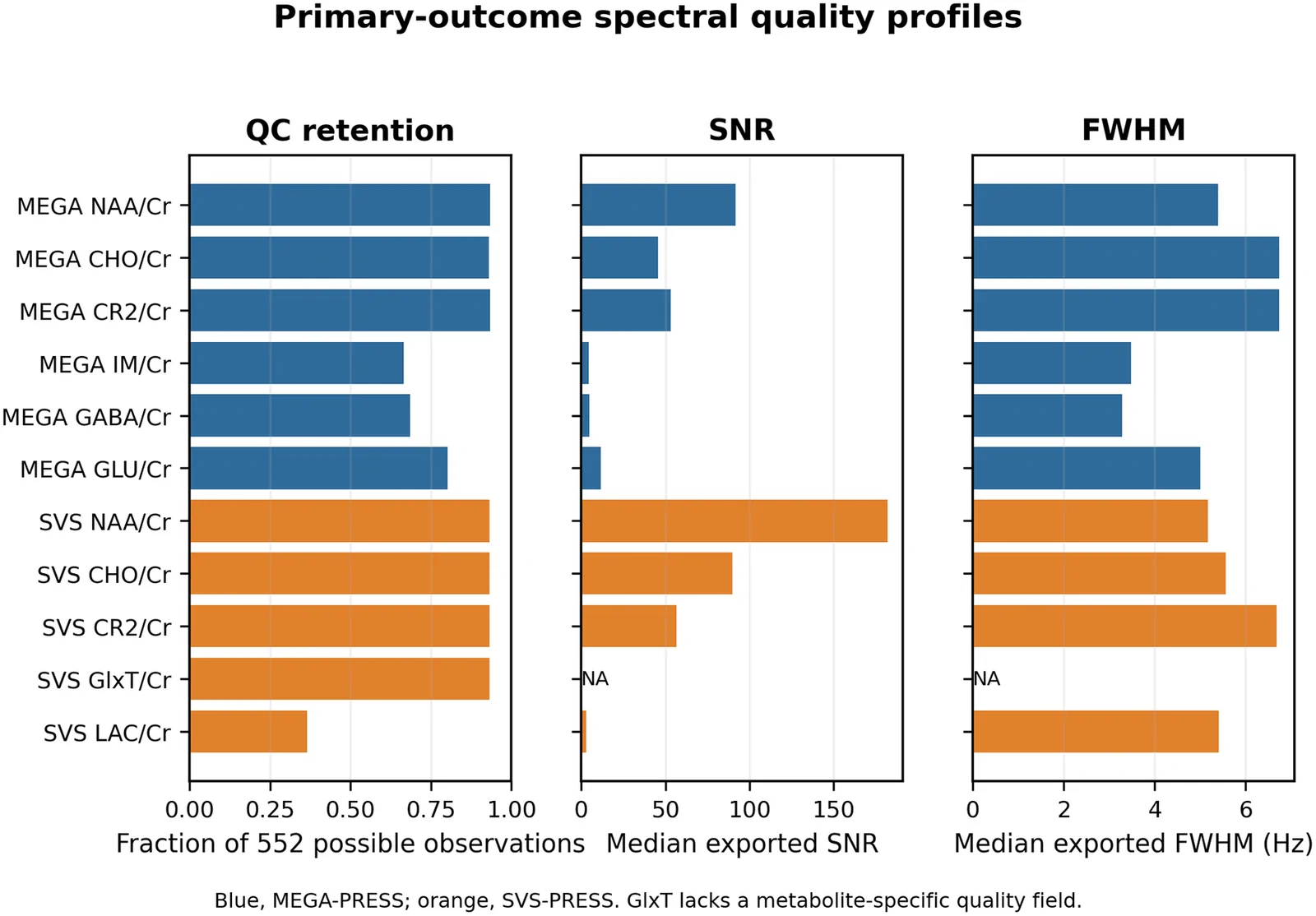
Primary-outcome spectral-quality profiles. Panels summarize retention under the reference QC rule (finite ratio, numerator SNR ≥ 3 and FWHM ≤ 13 Hz, and Cr SNR ≥ 3 and FWHM ≤ 13 Hz), median exported SNR, and median exported FWHM for the 11 primary sequence-specific outcomes among 138 participants. Retention is relative to 552 possible region-by-time-point observations per outcome. GlxT lacked a metabolite-specific quality field and therefore has no SNR or FWHM bar. Quality metrics are descriptive and denominators vary.

### Reference-metabolite and derived-axis analyses

Alternative NAA- and Cr2-referenced analyses largely preserved the regional hierarchy but also exposed denominator dependence (Figure 7; Supplementary Tables). ACC-weighted Cho/NAA and Cho/Cr2 supported a membrane-neuronal and membrane-energy contrast, whereas PCC-weighted NAA/Cho described the reciprocal relation. MEGA Glu/NAA and Glu/Cr2 remained ACC-weighted. The reciprocal overnight directions of SVS GlxT/NAA and NAA/GlxT were consistent with the primary GlxT/Cr reduction. Because all quantities remained ratios, these analyses clarify internal consistency but cannot identify whether a numerator, denominator, or both changed in absolute concentration.

**Figure 7:**
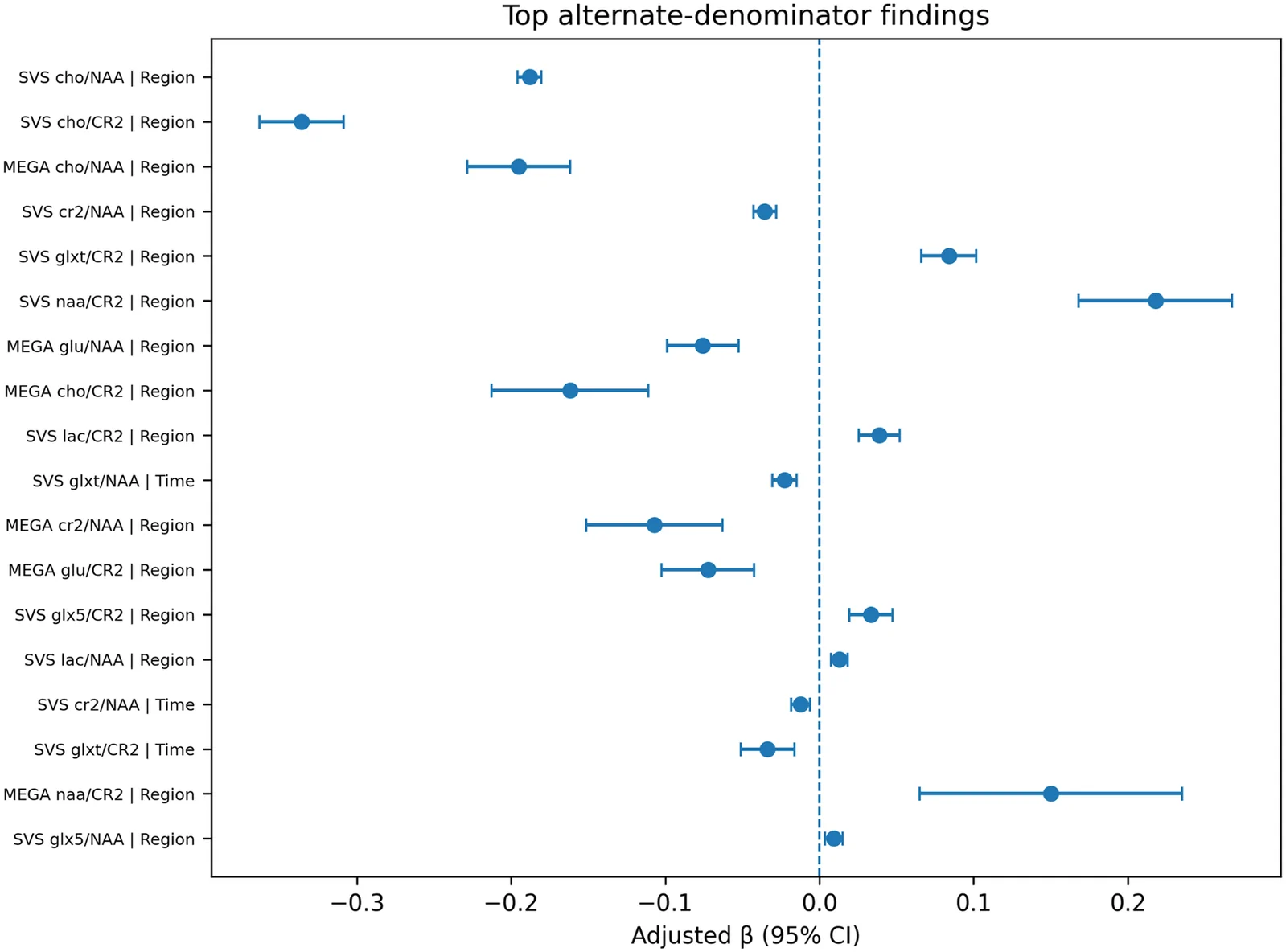
Selected alternative-reference findings. Forest plots show adjusted regional or time-point coefficients and 95% confidence intervals for NAA- and Cr2-referenced ratios (raw-scale models, *n* = 137). Alternative ratios test denominator sensitivity but do not represent absolute metabolite concentrations.

### Quality-control, selection, and PSG-adjusted sensitivity

Each robustness analysis addressed a distinct potential source of instability (Table 4). Across 16 QC specifications, all 10 estimable regional effects preserved their reference direction; eight of 10 time-point terms and eight of 10 interactions were fully direction-stable (Figure 8). QC-selection weighting preserved 11 of 12 targeted signs, and post-availability weighting preserved all 11 temporal directions. Excluding AHI ≥ 15 events/h (103 eligible participants) preserved the direction of all 12 primary FDR-significant effects, with 11 remaining *q* < 0.05; 32 of 33 region, time-point, and interaction directions matched the primary analysis. Excluding recorded hypertension or diabetes (123 eligible) preserved all 12 significant-effect directions, with 10 remaining *q* < 0.05; the combined restriction (100 eligible) retained 11 of 12 at *q* < 0.05. The few direction mismatches across restricted samples involved near-zero, nonsignificant terms. In PSG dose-response models (*n* = 117–122), no association survived correction across 77 tests; none of the 14 focused Cr2/Cr and GlxT/Cr tests survived the focused correction. Thus, measured sleep architecture, continuity, arousal, and respiratory burden did not provide corrected evidence for a simple dose-response explanation of the two temporal signals.

**Table 4:** Summary of robustness analyses.

Analysis	Target	Direction agreement	Scope or exception
QC threshold multiverse	Region	10/10 fully sign-stable	SNR 2–5 × FWHM 10–15 Hz
QC threshold multiverse	Time point	8/10 fully sign-stable	GlxT unavailable for multiverse
QC threshold multiverse	Interaction	8/10 fully sign-stable	Instability: weak mI/lactate terms
QC-selection IPW	GABA, Glu, mI, lactate	11/12 signs matched raw	Mismatch: mI interaction
Post-PSG availability IPW	All 11 temporal contrasts	11/11 signs matched unweighted	Weights based on demographics, AHI, baseline MRS
Expanded PSG adjustment	All primary terms	30/33 signs matched primary	All 12 primary FDR-significant terms remained *q* < 0.05
Positive-value log-ratio GEE	All primary terms	30/33 signs matched raw	Non-positive values excluded by definition
AHI restriction	All primary terms	32/33 directions matched	AHI <15 events/h; 11/12 primary FDR-significant terms remained *q* < 0.05
Recorded comorbidity restriction	All primary terms	32/33 directions matched	Hypertension and diabetes excluded; 10/12 primary FDR-significant terms remained *q* < 0.05
Combined restriction	All primary terms	31/33 directions matched	AHI <15 and no hypertension/diabetes; 11/12 primary FDR-significant terms remained *q* < 0.05
PSG dose-response	11 outcomes × 7 PSG measures	0/77 global FDR-significant	*n* = 117–122; 0/14 focused Cr2/Cr and GlxT/Cr tests survived focused FDR

**Figure 8:**
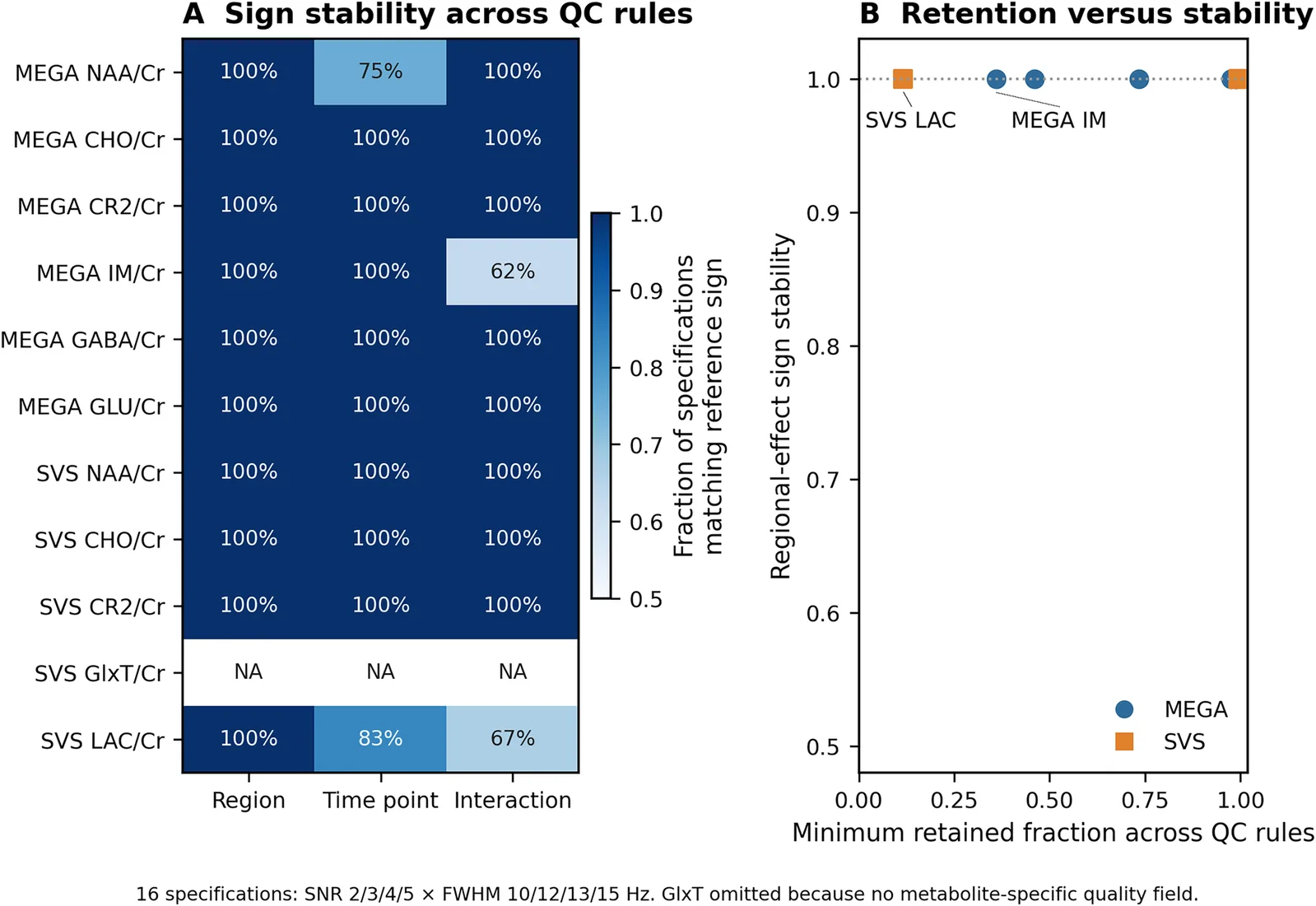
Quality-control multiverse. **(A)** Shows the fraction of SNR/FWHM specifications that preserved the reference effect direction; this sign stability does not imply statistical significance in every specification. **(B)** Relates minimum retained fraction to regional-effect direction stability. The 16 specifications crossed SNR cutoffs of 2, 3, 4, and 5 with FWHM cutoffs of 10, 12, 13, and 15 Hz. Across models, participant *n* ranged from 41 to 137 and retained observations from 59 to 514. GlxT lacked a metabolite-specific quality field.

### Exploratory clinical analyses

Exploratory phenotype analyses were materially less stable than the regional MRS results. Several nominal associations involved respiratory burden, mood, chronotype, memory, or NAA-, Cho-, and excitation-inhibition-related indices, but no MRS-clinical partial correlation survived the global correction. Cross-validated classification screens generated candidate depressive-symptom and eveningness models, but these were not externally validated and are not presented as diagnostic results. The coded smoking, alcohol, exercise, coffee, and tea variables showed no robust corrected associations. Full estimates remain in the supplementary workbook for transparency.

## Discussion

### Principal findings

This study provides quantitative support for a regional-first interpretation of paired cingulate MRS. The median absolute regional standardized effect was 4.6 times the median longitudinal effect; this is a ratio of effect-size summaries, not a biological fold change. Concordant NAA and Cho directions across sequences and the persistence of the main findings under proportional scaling, QC variation, selection weighting, and AHI/comorbidity restrictions make the regional pattern difficult to explain by one analytic choice. These analyses were complementary rather than redundant: standardized contrasts addressed magnitude, log models addressed scale, interaction models addressed sequence comparability, multiverse and weighting analyses addressed quality/availability selection, restriction analyses addressed cohort heterogeneity, and PSG regressions tested dose-response. The data do not show that longitudinal change is absent: SVS GlxT/Cr and Cr2/Cr decreased in the morning, but the design and null corrected PSG dose-response results do not identify sleep as their cause.

### Regional cingulate neurometabolic architecture

The anterior and posterior cingulate are functionally and chemically distinct. The ACC supports control, salience, and affective regulation, whereas the PCC anchors default-mode and memory-related processes (5, 6). Prior high-field work demonstrated cingulate gradients in GABA, glutamate, and glutamine, and a 3 T study identified an average rostral-to-caudal glutamate pattern with substantial individual heterogeneity (7, 8). Our higher ACC vendor-labeled MEGA Glu/Cr is directionally compatible with anterior predominance, while Cho/Cr was higher in ACC and NAA/Cr was higher in PCC across both sequences. However, these are acquisition-dependent ratios without tissue correction or absolute quantification. They therefore establish robust regional differences in measured metabolite ratios, not regional concentration gradients, cell counts, or metabolic fluxes.

The nominally macromolecule-suppressed GABA result requires particular care. PCC GABA/Cr was modestly higher at the baseline reference time point, while a region-by-time point interaction indicated differential change. Sleep duration and insomnia studies have reported ACC GABA associations, but repeated-measure findings are heterogeneous (15–18). The documented symmetric 1.90/1.50-ppm editing scheme supports macromolecule-suppressed rather than GABA + nomenclature (34, 35, 38). However, symmetric suppression is sensitive to frequency drift, and the retained exports did not include transient-level frequency traces or editing-pulse bandwidth and duration needed to verify suppression efficiency. We therefore interpret this as a nominally macromolecule-suppressed regional edited-GABA contrast rather than a pure neurotransmitter concentration difference. The same restraint applies to MEGA-derived Glu: agreement between Glu or Glx estimated from edited spectra and conventional PRESS can be poor and region-dependent (37).

### Longitudinal effects and the limits of a two-time-point design

The comparatively small longitudinal effects fit a literature in which neurochemical change depends on metabolite, age, sleep history, measurement time, and region (18–20). Post-PSG reductions in SVS GlxT/Cr and Cr2/Cr could reflect changes in numerator, denominator, or both. None of their focused associations with TST, sleep efficiency, WASO, slow-wave sleep, REM sleep, arousal index, or AHI survived FDR correction, providing no corrected evidence for a simple sleep-dose relationship. The verified schedule further narrows the appropriate interpretation: baseline MRS occurred on day 1 evening, PSG on the intervening second night, and follow-up MRS on day 3 morning, with a median scan-start interval of 34.01 h. This longitudinal contrast therefore combines elapsed time, circadian phase, the monitored night, an intervening day, fasting or feeding, time awake, scanner repositioning, and potential drift. The schedule was intended to preserve PSG sleep opportunity and avoid disruption of routine clinical scanning, and may have reduced acute pre-PSG burden from an evening MRI examination. However, the study had no same-night comparison arm, so it cannot establish biological equivalence between the implemented schedule and an immediate baseline-PSG-follow-up schedule or quantify any effect of the intervening day. A wake-control protocol, randomized acquisition order, and repeated within-sleep or circadian measurements would be needed to separate these components. The results are therefore described as longitudinal contrasts rather than sleep-caused changes.

Attrition sensitivity is relevant because the 12 participants without post-PSG spectra were older and had greater respiratory burden. Post-availability weighting based on demographics, AHI, and baseline MRS preserved every temporal direction and changed the two largest SVS estimates only modestly. This increases confidence under a missing-at-random model conditional on measured predictors, but it cannot resolve selection driven by unmeasured or missing post-PSG neurochemistry (47). The explicit missingness audit is more informative than silently treating all available observations as equally representative.

### Sequence and reference choice are part of the biological inference

NAA and Cho showed the same regional direction across sequences, yet interaction models demonstrated sequence-dependent regional magnitude for NAA and Cr2. MEGA-PRESS and conventional PRESS differ in echo time, editing, co-edited signal, relaxation weighting, baseline, and fitting behavior (10–12, 34–38). Directional replication is reassuring, but equality of coefficients should not be assumed and the sequences should not be pooled as interchangeable measurements. Nonsignificant sequence-by-time-point interactions likewise do not establish equivalence.

Reference choice creates a second layer of dependence. Creatine referencing was necessary because the retained exports did not contain validated absolute water-referenced concentrations, but Cr is not biologically fixed and can differ with region, tissue composition, or physiology. Replacing Cr with NAA or Cr2 preserved several directional conclusions while altering others, exactly as expected when both numerator and denominator vary (12, 39, 40, 43). The log-ratio analysis improved proportional interpretation and reduced scale dependence but did not solve reference confounding. Future work should retain unsuppressed water, relaxation information, tissue fractions, and raw transients and should report water-referenced, tissue-corrected concentrations alongside ratios rather than presenting alternative denominators as substitutes for absolute quantification.

### Quality control as a robustness dimension

Spectral QC is often reduced to one exclusion cutoff, although SNR and linewidth jointly affect precision, bias, and retention. Our threshold multiverse asked whether conclusions persisted across reasonable operational rules rather than declaring one cutoff uniquely valid. Every estimable regional direction was preserved; instabilities were confined to weak mI and lactate time-point or interaction terms. Sleep/wake-dependent lactate dynamics have been demonstrated in rodent cortex (49, 50), but these findings do not directly validate MRS-derived Lac/Cr in our human cohort. Selection weighting led to the same qualitative conclusion: strong regional effects persisted, whereas weak secondary terms were more sensitive. Sign stability here means consistency of effect direction and does not imply statistical significance under every QC specification.

Excluding low-quality spectra can reduce measurement error but can also select on region, time point, age, motion, anatomy, or disease burden. Reporting raw, thresholded, multiverse, and weighted estimates makes that dependence visible. Nevertheless, SNR and FWHM are incomplete: the retained exports lacked fit residuals, Cramer-Rao-type uncertainty, transient-level drift, water-suppression efficiency, and visual-rating fields. The QC analyses therefore assess the available metrics and do not constitute complete validation of spectral quantification.

### Clinical interpretation and biomarker restraint

The regional architecture was more reproducible than the broad MRS-clinical association screen. A stable group-level contrast is not automatically an individual diagnostic marker, particularly in a clinically heterogeneous cohort. Global correction removed the partial-correlation findings, and classifiers were developed and evaluated within one cohort. Mood, chronotype, cognition, memory, and respiratory burden are influenced by medication, circadian phase, sleep continuity, and measurement reliability. The main contribution is therefore a measurement framework—region separation, sequence-specific inference, reference sensitivity, and QC transparency—rather than a diagnostic cutoff or a phenotype-specific biomarker.

### Strengths, limitations, and next steps

Strengths include the prospective paired longitudinal design, full-night PSG, two cingulate regions, two spectroscopy sequences, a relatively large cohort for repeated MRS, participant-level effect-size comparison, formal sequence interactions, and sensitivity analyses addressing quality, attrition, respiratory burden, recorded cardiometabolic comorbidity, and PSG dose-response. The bootstrap hierarchy directly addressed the primary question without relying on counts of significant *P* values.

Several limitations constrain interpretation. The study was single-center, used a university-affiliated and advertisement-recruited sample, lacked independent replication and a matched wake-control protocol, and had complete paired timestamps for 116 of 138 participants; missing timestamps were not imputed. Among those 116 participants, all follow-up examinations occurred two calendar days after baseline and the median scan-start interval was 34.01 h. This improves timing transparency but does not isolate sleep, because the schedule included an intervening daytime period before the PSG night. Although this pragmatic schedule protected PSG sleep opportunity and routine clinical scanner availability, it cannot be assumed to be biologically equivalent to a same-night baseline-PSG-follow-up design. ACC was always acquired before PCC and the within-session order was fixed (ACC SVS-PRESS, ACC MEGA-PRESS, PCC SVS-PRESS, PCC MEGA-PRESS); region and elapsed acquisition time therefore cannot be fully disentangled. Screening totals, grouped exclusion reasons, aggregate educational categories, eligibility criteria, and fixed order were recovered from study records, but mutually exclusive reason-specific counts for the 12 exclusions were unavailable. Fourth-year interns were not assigned night-shift rotations, but not all participants in the undergraduate or bachelor's-level category were interns, and individual internship status was not fully resolved. Night-shift or on-call exposure was not systematically collected for the full cohort; it was not inferred from education or recruitment category, and no duty-status subgroup analysis was performed. Relevant medication use and known sleep disorders were exclusion criteria, but no analyzable participant-level medication inventory remained. One in-laboratory PSG night may be affected by a first-night effect, consistent with mean sleep efficiency of 84% and TST of approximately 6 h (51). Voxel repositioning was guided visually by the same operator but not quantified with overlap maps. Tissue segmentation, partial-volume correction, relaxation correction, raw transients, an independently acquired unsuppressed-water reference, fit residuals, editing-pulse bandwidth/duration, and validated absolute concentrations were unavailable. The vendor model internals were not exposed. SVS-PRESS TE 144 ms limits J-coupled-metabolite quantification, and edited signals remain sensitive to macromolecular suppression, drift, and model dependence. The operational QC metrics were incomplete, and extensive phenotype analyses remain exploratory.

Future studies should preregister a small primary metabolite set; retain reason-specific screening counts, individual recruitment and duty schedules, complete scan timestamps and intervals, participant-level medication records, individual transients, unsuppressed water, and fitting diagnostics; quantify voxel overlap and tissue fractions; and use tissue- and relaxation-corrected water-referenced estimates alongside ratios. A matched wake-control condition or repeated circadian sampling is required for sleep-specific causal inference. Harmonized acquisition and processing across vendors would test whether the regional hierarchy generalizes beyond one platform (39, 40, 52–56). External cohorts should evaluate clinical models without feature reselection.

## Conclusion

In this prospective paired cingulate-MRS cohort, ACC-PCC differences in metabolite ratios were substantially larger and more robust than longitudinal changes across the approximately 34-h protocol. Regional NAA/Cr and Cho/Cr directions were reproduced across sequences, while sequence interactions showed that MEGA-PRESS and SVS-PRESS were not interchangeable. The longitudinal findings were concentrated in SVS GlxT/Cr and Cr2/Cr, but no corrected PSG dose-response association identified sleep architecture or quality as their driver. Because the measurements were Cr-referenced, not tissue-corrected, and not absolutely quantified, the results support a regional-first, sequence-specific, reference-aware framework for cingulate MRS rather than claims of regional concentration differences, sleep-specific causation, or individual biomarkers.

## Data Availability

The datasets presented in this article are not readily available because participant privacy and institutional data-governance requirements restrict public sharing. De-identified data may be made available on reasonable request, subject to ethics approval and institutional policy. Requests to access the datasets should be directed to Fajin Lv, fajinlv@163.com; Yida Hu, hyd1112@126.com.

## Supplementary material

SUPPLEMENTARY FILE 1 Expanded statistical tables for the accepted manuscript, including cohort characteristics, primary and sensitivity analyses, exploratory analyses, data-cleaning and acquisition-missingness audits, and scan-timing summaries.
